# Hybrid Deep Learning Models for Analyzing Histological Images of the Zebrafish Intestine Under Oxidative Stress

**DOI:** 10.3390/diagnostics15222859

**Published:** 2025-11-12

**Authors:** Cristian Dan Pavel, Simona Moldovanu, Irina Andreea Pavel, Oana-Maria Dragostin, Carmen Lidia Chițescu, Carmen Lăcrămioara Zamfir

**Affiliations:** 1 Department of Morphofunctional Sciences I, Faculty of Medicine, Grigore T. Popa University of Medicine and Pharmacy, 700115 Iasi, Romania; cristian-dan_g_pavel@d.umfiasi.ro (C.D.P.); carmen.zamfir@umfiasi.ro (C.L.Z.); 2 Department of Computer Science and Information Technology, Faculty of Automation, Computers, Electrical Engineering and Electronics, Dunarea de Jos University of Galati, 800146 Galati, Romania; simona.moldovanu@ugal.ro; 3 The Modelling & Simulation Laboratory, Dunarea de Jos University of Galati, 47 Domneasca Str., 800008 Galati, Romania; 4 Department of Surgery II, Faculty of Medicine, Grigore T. Popa University of Medicine and Pharmacy, University Street, No.16, 700115 Iasi, Romania; 5 Research Centre in the Medical-Pharmaceutical Field, Faculty of Medicine and Pharmacy, Dunarea de Jos University of Galati, 800201 Galati, Romania; oana.dragostin@ugal.ro (O.-M.D.); carmen.chitescu@ugal.ro (C.L.C.)

**Keywords:** oxidative stress, zebrafish, intestine, methylxanthines, theobromine, caffeine

## Abstract

**Background/Objectives**: Convolutional Neural Networks (CNNs) and advanced image pre-processing can enhance the classification of antioxidant effects applied on zebrafish intestine. This study proposes a hybrid technique that combines four deep learning (DL) models: the pre-trained Xception CNN, a custom-built autoencoder, a custom-built CNN, and a Vision Transformer (ViT). **Methods**: For classification features generated by DL models, the Support Vector Machine (SVM), Random Forest (RF), and k-Nearest Neighbors (kNN) algorithms were proposed. Contrast-Limited Adaptive Histogram Equalization (CLAHE) and the mentioned DL artificial intelligence (AI) algorithms were applied to improve the accuracy of the classification of histological images of zebrafish intestinal morphology. **Results**: In a binary classification, the following classes were studied on zebrafish intestine: (i) control and experimental-induced oxidative stress (OS); (ii) OS versus OS and theobromine (TB); and (iii) OS versus OS and caffeine (CAF). The novelty of the research lies in applying CLAHE to enhance image quality and utilizing four hybrid models to improve classification accuracy compared to raw images, when a private dataset of zebrafish intestine histology under certain chemical treatments (OS, TB, CAF) was employed. **Conclusions**: The best results are obtained in a binary classification with a hybrid combination of Xception and SVM for OS versus OS and TB classes, with an accuracy of 84.6% for pre-processed images, better than raw images, when the accuracy was 78.4%.

## 1. Introduction

OS arises, according to its classical definition, when the production of reactive oxygen species (ROS) exceeds the capacity of cellular antioxidant defenses, leading to oxidative damage of lipids, proteins, and nucleic acids [1]. This imbalance is a central and very complex mechanism in the pathogenesis of inflammatory processes, different types of metabolic dysfunctions and gastrointestinal disorders across vertebrates. Different types of biomarkers, such as lipid peroxidation products, or antioxidant enzymes, such as superoxide dismutase, catalase, and glutathione peroxidase, are used to assess the oxidative status. The various effects of oxidative stress on different organs can generate extremely complex pathologic processes [2].

The zebrafish has earned its place among viable models for the study of oxidative stress due to its genetic accessibility and embryonic transparency that facilitate real-time imaging and conserved molecular pathways of redox regulation [3]. Studies have shown that different types of environmental toxicants, including microplastics, nanoparticles, and antibiotics, induce oxidative stress in zebrafish intestine, with all the consequences that come from distinct and pronounced alteration in intestinal redox balance, impaired microbiota structure, and are correlated with specific histopathological changes in the intestinal mucosa, especially affecting the epithelial component [4,5]. Collectively, these findings sustain the zebrafish intestine as a relevant and sensitive target of oxidative injury and support its value as a reliable in vivo system for identifying, characterizing, and interpreting the contribution of oxidative stress to intestinal pathophysiology [6].

In addition to pollutants, a significant number of dietary and pharmacological compounds can modulate oxidative outcomes [7]. Among them, theobromine and caffeine are known for their dual antioxidant/oxidizing potential, depending on concentration and context. Members of the same family of methylxanthines, they exhibit distinct effects under oxidative stress. Caffeine, acting as an adenosine receptor antagonist and phosphodiesterase inhibitor, stimulates metabolism and increases the production of reactive oxygen species (ROS). Theobromine, with a lower affinity for adenosine receptors and phosphodiesterase, will induce a lower metabolic effect, but significant antioxidant activity. The inflammatory mediators are reduced by theobromine, due to its potential to inhibit the Nf- κB pathway; as a result, pro-inflammatory cytokines decrease. Another pathway, Nrf2, can also be activated by theobromine, stimulating the expression of antioxidant enzymes and increasing cellular defense against oxidative damage. These aspects support its anti-inflammatory and antioxidant activities, which contrast with caffeine’s primarily metabolic and pro-oxidant effects [8]. Usually, high doses correlate with the initiation of oxidative stress, while moderate doses protect against oxidative alterations [9]. In zebrafish larvae, dietary antioxidants have been shown to mitigate inflammation and oxidative damage, demonstrating the suitability of this model for in vivo pharmacological testing [10,11].

On the one hand, considerable research has examined the role of methylxanthines in neurodegenerative condition, diabetes, respiratory diseases and cancer. On the other hand, the reduced number of studies regarding the effect of methylxanthines on oxidative stress in the intestine supports a more careful evaluation and interpretation of their role as modulators or inducers of oxidative alteration at this level.

Classifications of histologic images are new trends in translational medicine and clinical practice. Typically, the classification process involves several stages: data pre-processing, implementing a custom-built CNN, using pre-trained CNNs, and applying the transfer learning process. Additionally, the DL workflow includes training and enhancing network performance by adjusting hyperparameters, conducting multiple trials through the ablation method, and visualizing as well as monitoring network activity during and after training.

On intestinal histological images, the scientific literature is not so extensive, with more authors proposing particular DL models applied on public datasets or pre-trained models having different architectures. A deep neural network named GasMIL was proposed by Fang et al. [12], which showed performance in diagnosing intestinal metaplasia. The EfficientNet-b0, DenseNet-201, ResNet-101, MobileNet-v2, and Xception pre-trained CNNs were included in the study proposed by Ibrahim et al. [13] for distinguishing between H. pylori-positive and H. pylori-negative cases, classifying 204 histopathological images. Liu et al. [14] studied Crohn’s disease and intestinal tuberculosis, applying deep learning algorithms to analyze whole slide images of surgical specimens to distinguish the studied classes. The Cancer Genome Atlas has histological images, clinical data, and an abundance of molecular data. With this information, Li et al. [15] conducted a full analysis, including clinical variables, gene expression, and imagery features taken from a hematoxylin and eosin image with a residual network (Resnet).

The pre-trained CNN models used in previous studies enable transfer learning, which means the models can apply what they learned in one area to assist with another task. The histological images collected from the intestine of zebrafish represent a novelty in the DL field, so, in addition to a pre-trained CNN, the paper also proposes a custom-built autoencoder and transformer models. Comprehensive experiments are presented, and a full comparison analysis is conducted to evaluate the models’ performance and limitations.

Many studies omit the pre-processing of images; however, it has been shown that incorporating this stage can yield significant results that surpass those achieved with raw data. This enhancement not only improves the accuracy of the models but also helps in reducing noise and irrelevant features that could hinder performance. As a result, more researchers are beginning to acknowledge the importance of pre-processing in their methodologies. The pre-processing CLAHE method enhances image quality by improving local contrast. This makes it a valuable pre-processing technique for a variety of applications, such as medical image analysis and feature extraction [16,17].

In this sense, our main contributions can be summarized as follows.

(1) The acquisition of histopathological images of the intestine includes the following classes: control, induced OS, OS and TB, and OS and CAF. This was performed using a Zeiss Axiolab 5 microscope and an Axiocam 208 color camera(Carl Zeiss Microscopy GmbH, Jena, Germany.).
(2) Four distinct DL architectures on a novel zebrafish intestinal histology dataset, demonstrating that a hybrid Xception-SVM pipeline on CLAHE-enhanced images achieves state-of-the-art performance for this specific biological context, were constructed and benchmarked.
(3) The best information in corroboration with the relationship between deep learning models and studied classes was discovered. Using pre-processed images prior to training the deep learning model was made advantageous by the primary finding. By integrating these insights, we refined our approaches and enhanced the overall effectiveness of their models.
(4) The classification process by providing the deep learning models with images that were pre-processed using the CLAHE method was enhanced. This allowed for improved contrast in the images, enabling the models to better identify subtle features.
(5) The performance of the models in terms of accuracy, F1-score, and MCC was expressed. These metrics provide a comprehensive view of how well the models are performing across different aspects, allowing us to identify strengths and weaknesses in their predictions.

The following sections of this work are organized as follows:

Section 2 describes the methodology and resources utilized in this work to examine images of zebrafish intestine in different experimental conditions. Section 3 and Section 4 provide an explanation of the results and subsequent discussions. Section 5 concludes the study.

## 2. Materials and Methods

**Figure 1:**
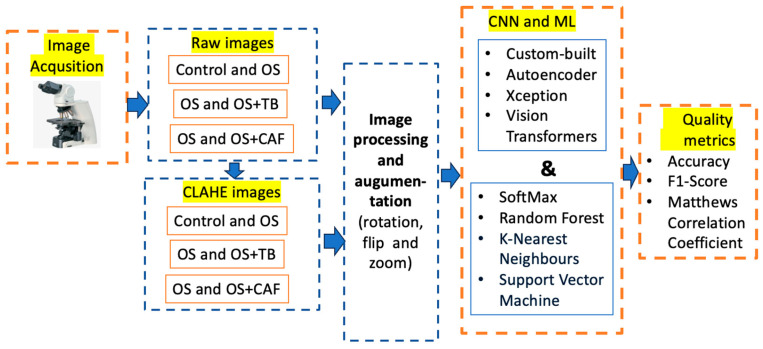
The workflow of the proposed study.

The raw images were acquired using a Zeiss Axiolab 5 microscope (Carl Zeiss Microscopy GmbH, Oberkochen, Germany) (first block) from the intestines of zebrafish exposed to induced OS, and OS associated with methylxanthines–theobromine and caffeine. The input images were categorized (control, OS, OS and TB, OS and CAF) in both raw and pre-processed (second block). The pre-trained and custom-built CNNs were provided with both raw photos and enhanced images utilizing CLAHE techniques. All images were resized to the dimensions compatible with the pre-trained CNN, and three augmentation methods were employed to enhance classification (third block). Four distinct deep learning models, namely Xception, autoencoders, custom-built CNNs, and Vision Transformer, were proposed for feature extraction and large-scale vision tasks, applicable to limited datasets (the fourth block). The classification process was carried out using SoftMax, Random Forest, K-Nearest Neighbors, and Support Vector Machine methods. The classification procedure was assessed using accuracy, F1-score, and Matthews Correlation Coefficient (MCC) quality measures. All enumerated stages are shown in Figure 1.

The following subsection provides a detailed discussion of all the blocks mentioned in this methodology. Each block will be analyzed in terms of its function, significance, and interconnections with the other components within the framework. This comprehensive examination aims to clarify how each method contributes to the overall effectiveness of the methodology.

### 2.1. Dataset

The proposed CNNs were trained using an image database containing 1845 control instances, 1305 instances of oxidative stress, 1188 instances of oxidative stress combined with theobromine and 1107 instances of oxidative stress combined with caffeine, after the augmentation process. The original biological samples were 230 control instances, 145 OS, 132 OS and TBR, 123 OS and CAF.

Each experimental group included 10 zebrafish individuals, with 4 intestinal sections examined per specimen. From each section, 3–5 representative microscopic fields of view were selected for further processing, depending on tissue integrity and the presence of well-defined histological features. Non representatives or artifact-containing fields were excluded from the dataset. Images were acquired at 10× magnification. Train/test splits were performed at the animal level.

The oversampling process is unnecessary because the dataset is moderately imbalanced; the percentages are calculated based on a classification of the classes: (i) control 58.5% and OS 41.5%; (ii) OS 52.3% versus OS and TB 47.7%; (iii) OS 54% versus OS and TB 46%.

**Figure 2:**
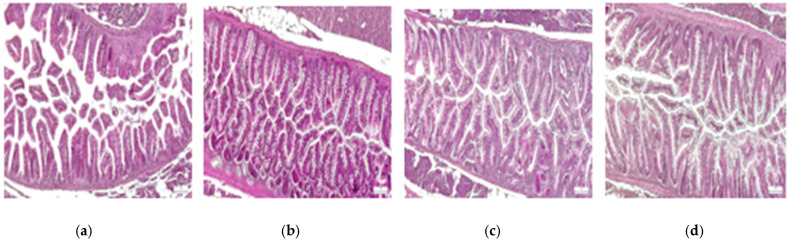
Samples form proposed dataset: zebrafish intestine. (**a**) control; (**b**) OS; (**c**) OS and TB; (**d**) OS and CAF. HE staining, 10×; scale bar, 100 μm.

The original images were in RGB channels, and the pre-processed image was with CLAHE in grayscale. The size of images was established at 299 × 299 for Xception, 72 × 72 for ViT, and 250 × 250 for custom-built and autoencoder CNN. Figure 2 depicts samples from control, oxidative stress, oxidative stress and theobromine, and oxidative stress and caffeine histological images.

**Figure 3:**
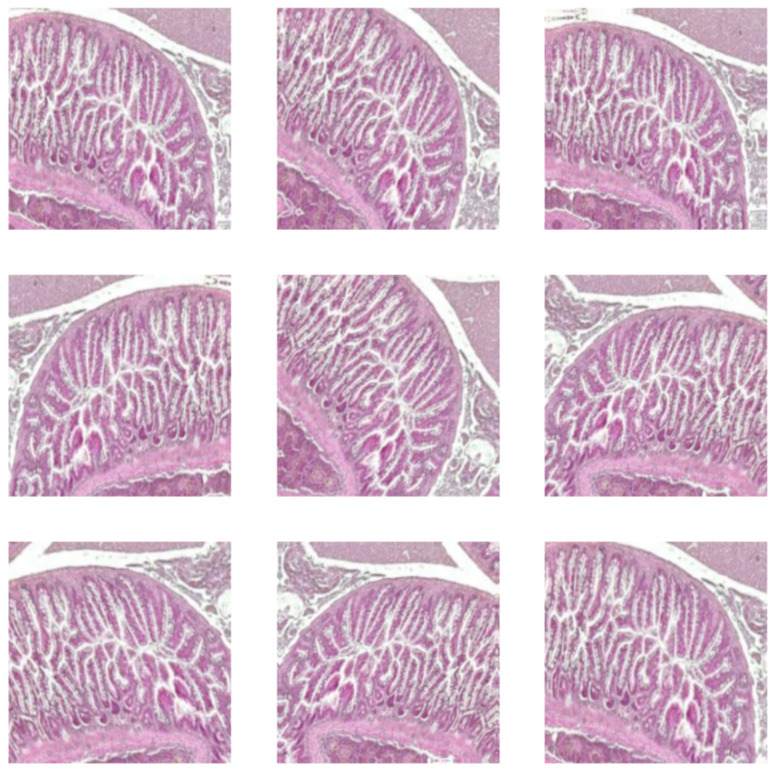
Augmented intestine histological images.

The augmentation incorporates random rotations up to 10 degrees, random zooms with 10% interpolated pixels, and random horizontal flips with a 10% probability. A sample of augmentation in Figure 3 is shown. This technique was applied to the training dataset, which included both raw images and images that were pre-processed using the CLAHE method. It was necessary because it enhanced the DL model’s ability to generalize by introducing variability in the training data. As a result, the model becomes more robust and for classification intestine histological images.

### 2.2. Hardware and Software

The hardware architecture consists of a PC with an Apple Mac Studio (2022), an Apple M1 Max, a 1 TB SSD, 32 GB, and a 32-core Apple GPU. The minimization of run time was performed in the Google Collaboratory environment with the T4 GPU hardware accelerator.

The training and testing of AI algorithms in the software environment utilized Python (3.12), scikit-learn (1.5.2), TensorFlow (2.18.0), Keras (3.6.0), and Visualkeras (0.1.4.3). The CLAHE pre-processed images by using MATLAB 2021a (The MathWorks, Natick, MA, USA) and the Image Processing Library were performed.

The proposed dataset is imbalanced; to handle this imbalance during training, the focal loss function was used. Besides predicted outputs and ground truth labels parameters, the weighting factor alpha of 0.25 and a focusing parameter gamma of 2 were used. The proposed models in are stored in a repository with its web address (https://github.com/simonamoldovanu/zebrafish, accessed on 25 October 2025).

### 2.3. Convolutional Neural Network and Machine Learning Algorithms

#### 2.3.1. Xception

**Figure 4:**
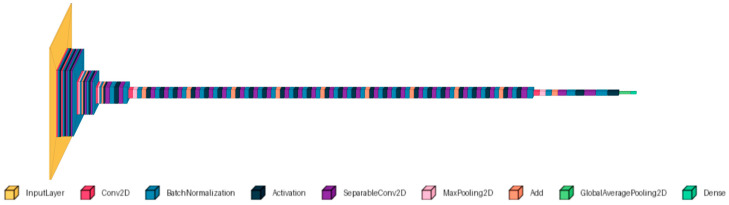
The Xception architecture.

The pre-trained Xception is an Inception-inspired convolutional network that replaces Inception modules with depthwise separable convolutions. The model is trained on the ImageNet database, which contains images with a default input size of 299 × 299 pixels and three-color channels [18]. This CNN was applied to breast [19], colorectal cancer [20], and lung and colon [21] histologic images. These papers demonstrate the Xception’s versatility and effectiveness in medical imaging tasks. Additionally, the adaptability of Xception allows it to be fine-tuned for various other datasets, making it a valuable tool for our dataset. The used architecture is shown in Figure 4. The extracted features from Xception (with frozen base) were used with separate classifiers: SVM, RF, and KNN.

#### 2.3.2. Custom-Built CNN

**Figure 5:**
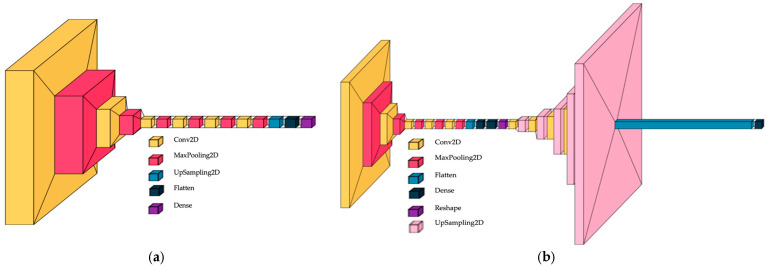
CNN architectures; (**a**) custom-built CNN; (**b**) convolutional autoencoder CNN.

We proposed a custom-built CNN that includes six convolutional layers, six max pooling layers, one upsampling layer for 2D inputs, and one flattened layer, followed by one dense layer. The final architecture was established after modifying the initial design during the ablation process, which involved increasing the number of convolutional layers from one to ten; the optimum model is illustrated in Figure 5a. The ablation process involved adjusting the number of convolutional layers and neurons. The architecture of the custom-built CNN was designed with 4, 5, 6, and 7 layers, and the number of neurons doubled starting from the first to the next. For each architecture, the classification accuracy was computed. The best accuracy was achieved with the six-layer architecture, and the variation in accuracy among the models was approximately 0.02%. The input size was 250 × 250 and three channels. The proposed study, which contains a specific dataset of histologic images, led to the development of a custom-built CNN as a solution.

#### 2.3.3. Convolutional Autoencoder

A convolutional autoencoder can interpret the complex pattern of histologic images. Different CAE architectures were tested in the paper proposed by Munteanu et al. [22], where the symmetric encoder–decoder with bottleneck layers gave salient results, so we continued the idea, and the architecture with the following structure was proposed: five convolutional layers for the encoder and decoder, respectively; six max pooling layers; five upsampling layers for 2D inputs; one flattened layer; and one dense layer for the classification process. The bottleneck has two dense layers: one flattens, and one reshapes for simultaneous nucleus detection and feature extraction in histopathology tissue images. For the autoencoder CNN, the ablation process consisted of the creation and testing of the architectures: (i) removing the bottleneck; (ii) removing the first convolutional layer from the encoder; and (iii) adding the convolutional layer from the encoder and removing the last layer from the decoder. The accuracy of classification was higher by 0.03% for the symmetric encoder–decoder with bottleneck layers compared with other models. The autoencoder architecture in Figure 5b is shown. The input size was established to 250 × 250 and three channels. Hou et al. proposed a salient model for detection and feature extraction in histologic tissue images [23].

#### 2.3.4. Vision Transformer

**Figure 6:**
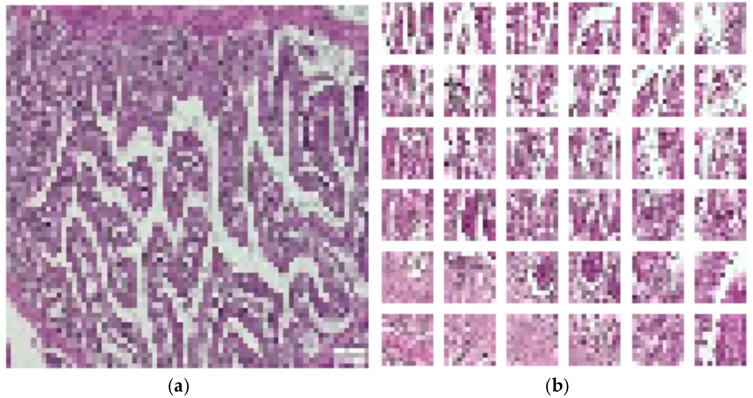
Input for ViT deep learning model. (**a**) Pre-processed image. (**b**) Generated image patched.

ViT is a deep learning model for computer vision, designed as an alternative to CNNs. The core of ViT consists of encoding with an encoder and a classification head, which take images divided into patches as input. In our study, the images were resized to 72 × 72 pixels, with a patch size of 12 × 12 pixels, resulting in 36 patches per image and 432 elements per patch. Various ViT architectures were developed to outperform convolutional neural networks [24]. These architectures were tested based on their ability to accurately classify and analyze the detailed features present in histological images. Preliminary results suggest that ViT could offer enhanced performance, especially in detecting subtle patterns that traditional CNNs might miss. Figure 6 depicts the resized and divided images in patches for ViT deep learning model.

#### 2.3.5. Machine Learning Classifiers

SoftMax is employed in CNNs to transform the last layer logits into probability distributions. This enables the model to provide predictions by specifying the probability of each class. When the application of the SoftMax function does not enhance decision-making based on expected probability, the hybrid CNN and ML model achieves the highest accuracy. The most common MLs are RF, kNN, and SVM, because they are adequate for small databases, robust to noise, and effective for high-dimensional CNN features [25]. In the proposed study, in the last layer of CNN in classification tasks, SoftMax and RF, kNN, and SVM were applied. Researchers proposed a hybrid approach that combines MobileNet-VGG16, VGG16-AlexNet, and MobileNet-AlexNet with XGBoost algorithms and decision tree (DT) for analyzing histopathological images of malignant lymphomas [26]. Breast cancer detection from histopathology images taken using a hybrid model is presented in [27], where the authors use ResNet50 and VGG16 for feature extraction and Extra Tree Classifier, Logistic Regression, Voting Classifier, Ridge Classifier, and SVM.

The SVM tends to perform well with features extracted by deep learning (DL) because it can identify optimal separating hyperplanes in high-dimensional spaces. However, kNN and RF may not perform as effectively for several reasons. kNN depends on distance metrics, which can be problematic when dealing with high-dimensional features in DL. On the other hand, RF constructs decision trees based on feature thresholds to split data, which can result in overfitting or suboptimal performance, particularly when the number of features greatly exceeds the number of training samples.

#### 2.3.6. Contrast-Limited Adaptive Histogram Equalization

This study utilized CLAHE (Contrast-Limited Adaptive Histogram Equalization) processing technology to significantly enhance the contrast of the images and accentuate the edges throughout. This enhancement improves the visibility of features that may have been obscured in the original images. The core concept of CLAHE (Contrast-Limited Adaptive Histogram Equalization) is to perform histogram equalization on non-overlapping segments of an image. After this process, bilinear interpolation is applied to address any edge-related issues. Bilinear interpolation merges the tiles around the edges to eliminate artificial borders and is a fundamental resampling method employed in image processing and computer vision [28]. Each image from the control group, as well as those from the oxidative stress, oxidative stress and theobromine, and oxidative stress and caffeine groups, were enhanced, resulting in a flat histogram with a contrast enhancement clip limit of 0.02.

**Figure 7:**
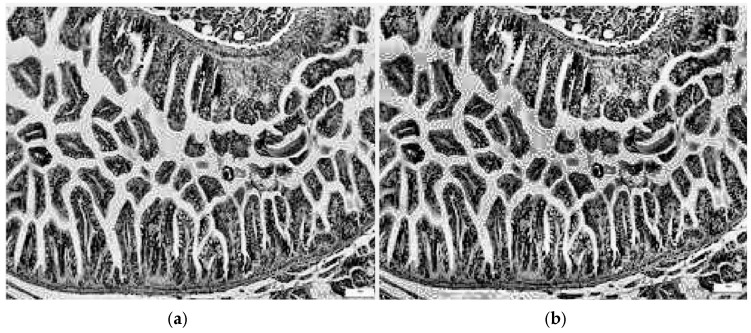
Image processing utilizing the CLAHE approach: (**a**) grayscale image; (**b**) image with modified contrast.

Equations (1)–(4) describe the CLAHE computer vision method. Additionally, a sample from the control class is presented, highlighting modified contrast along with the corresponding histograms shown in Figure 7.

Each grayscale image *I*(*x*,*y*) shown in Figure 7a was resized at dimensions compatible with proposed CNNs, where each tile *T*(*i*,*j*) has its own histogram:

(1) $$h_{i,j}\left(k\right)=\sum_{\left(x,y\right)\in T_{i,j}}\delta \left(I\left(x,y\right)-k\right),\;k\in \left\{0,1,\ldots ,L-1\right\}$$

where *L* is the number of intensity levels and *δ*(⋅) is the Kronecker delta.

Each histogram bin is clipped to a clip limit *C*.

(2) $$h_{i,j}^{\prime }\left(k\right)=min\left(h_{i,j}\left(k\right),\;C\right)$$

The total clipped excess is given by the following:

(3) $$E_{i,j}=\sum_{k=0}^{L-1}max\left(h_{i,j}\left(k\right)-C,0\right)$$

To prevent block artifacts, the new intensity of each pixel is derived by bilinear interpolation among the mappings of the four adjacent tiles.

For a pixel at coordinates (*x*,*y*) with fractional distances *α*, *β*∈[0, 1] to the surrounding tiles, a new *I*′ (*x*,*y*) image is obtained [29], a sample is shown in Figure 7b:

(4) $$I\prime \left(x,y\right)=\left(1-\alpha \right)\left(1-\beta \right)M_{i,j}\left(I\left(x,y\right)\right)+\alpha \left(1-\beta \right)M_{i+1,j}\left(I\left(x,y\right)\right)+\left(1-\alpha \right)\beta M_{i,j+1}\left(I\left(x,y\right)\right)+\alpha \beta M_{i+1,j+1}\left(I\left(x,y\right)\right)$$

The used parameters of CLAHE method used for pre-processing images were 8 × 8 tile size for more local contrast enhancement; with a clip limit of 0.2 moderate contrast enhancement, the contrast was redistributed in a uniform way.

#### 2.3.7. Ethical Approval

All experimental procedures involving zebrafish were approved by the Medical Ethics Committee of the “Gr. T. Popa” University of Medicine and Pharmacy, Iași, Romania umder protocol number 325/15 June 2023, in accordance with the institutional guidelines for the care and use of laboratory animals and complied with the ARRIVE GUIDELINES. All the zebrafish were euthanized through a gradual cooling method in crushed-ice–water, following the AVMA Guidelines for the Euthanasia of Animals (2020).

## 3. Results

**Figure 8:**
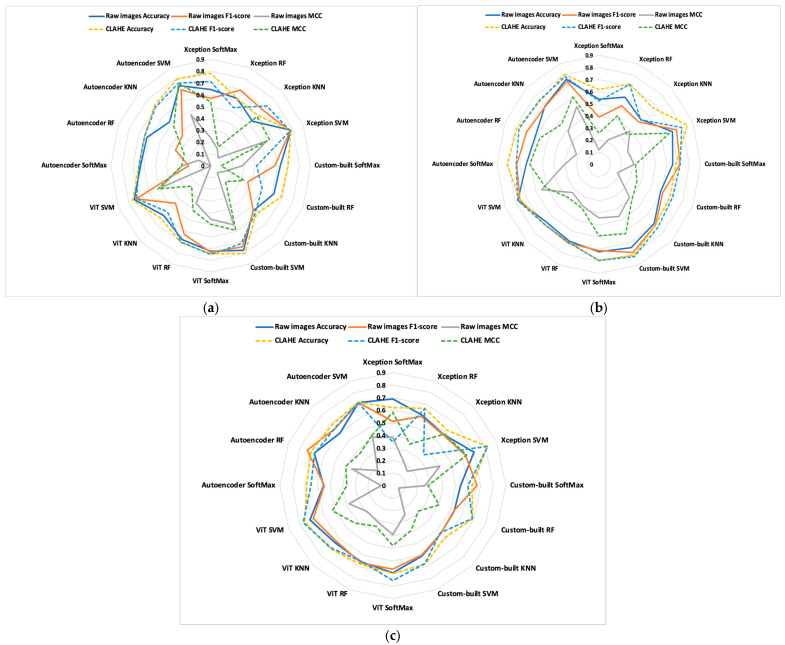
Comparisons of models in studied classes; (**a**) control vs. OS; (**b**) OS vs. OS and TB; (**c**) OS vs. OS and CAF.

Considering the previously outlined configuration of methodology in Figure 1, we achieved a high accuracy with a train–test split of 70–30%. The models and studied classes were evaluated with accuracy, F1-score, and MCC; these are defined as follows. Accuracy is the proportion of correctly classified images out of the total images evaluated. The F1 score is determined as the harmonic mean of precision and recall. When the data are unbalanced, the MCC metric must be computed as a measure of the quality of predictions over all samples of the confusion matrix. Different deep learning models with varying complexity levels are employed to balance accuracy against computational cost or to assess interpretability. Among the pre-trained CNNs, Xception was chosen because it demonstrates strong predictive performance on histologic images; additionally, a custom-built autoencoder and another CNN are trained to verify whether the original architecture can be applied to this type of image, while ViT represents a new generation of deep learning models. The evaluation was performed in two directions: (i) finding an adequate model for evaluating the predictability for proposed deep learning models expressed by metrics, as shown in Table 1, Table 2, Table 3 and Table 4; (ii) finding the higher differences between control, OS, OS and TB, and OS and CAF classes, as depicted in Figure 8.

The model showed a stronger ability to distinguish between the group with OS and those co-exposed to TB compared with CAF. This aspect reflects the real morphological differences in the intestinal response to these compounds. TB was associated with a better tissular preservation and improved antioxidant balance, while CAF provided a less pronounced protective effect. These findings are consistent with previous evidence that TB exerts a more protective antioxidant and anti-inflammatory responses than CAF at comparable concentrations, supporting the reliability of model’s results.

The classifier is generally utilized in the output layer of the neural network, particularly for classification tasks, to transform the raw output scores (logits) into a probability distribution [30]. To improve classification efficiency, the SoftMax function applied on the output layer was replaced with RF, kNN, and SVM. The classification process was performed on both raw and pre-processed images using the CLAHE method.

For training the DL models, the used hyperparameters were 20 epochs and a batch size of 16, and the resolution of images was established in accordance with the DL model.

From the results summarized into Table 1, Table 2, Table 3 and Table 4, we can clearly see the obtained best results. The discussions are made in accordance with the studied classes, ML algorithm image type and DL model.

In a binary classification, the best results are obtained for control vs. OS and OS vs. OS and TB. In this case, the OS and CAF do not influence much of the low-level features (edge, corners and texture) of the histopathological intestine.

All deep learning models are sensitive to pre-processed images with the CLAHE method. The average accuracy increases by 6.9% for Xception, by 6.4% for custom-built CNN, by 2.6% for ViT, and by 8.5% for autoencoder between raw and pre-processed images.

The explications are given by pre-processing images with CLAHE; this method enhances local contrast in small regions of the image, and edges become more pronounced because the local histogram equalization increases local contrast.

For all models, the metric quality values of the SVM classifier exceed those of SoftMax, KNN, and RF classifiers. The SVM effectively handles many features in transfer learning when it is applied together with Xception CNN, achieving an accuracy of 0.846 for OS versus OS and TB, when the network was supplied with images pre-processed using CLAHE. Also, for the same type of images and ML, the custom-built CNN provides an accuracy of 0.81. Weak results are obtained with ViT, even if, as in this case, the SVM gives good results compared with SoftMax, KNN, and RF classifiers.

In this paper, the best model was found for intestine histologic image classification for control, OS, OS and TB, and OS and CAF classes. Figure 8 shows each hybrid, CNN, and ML for the binary classifications of control vs. OS, OS vs. OS and TB, and OS vs. OS and CAF.

The comparisons enable us to gain new insights. To emphasize the differences in metric quality values, dashed lines represent pre-processed images, while solid lines indicate raw images. For all metrics, the most significant differences between the studied classes and proposed models occur between OS versus OS and TB. Additionally, the dashed lines indicate that the results obtained from pre-processed images are better than those obtained from raw images.

## 4. Discussions

Our experiments reveal three key characteristics that form the basis of our main findings:

The impact of pre-processing: A trend observed in our results indicated that increased pre-processing enhances classification quality, while raw images lead to decreased performance. This finding is consistent across all the deep learning models we have utilized. Additionally, cutting-edge papers proposed other image types with the same pre-processing method.

Hadiyoso et al. [31] reported the best results on a public dataset containing lung and colon cancer histopathological images, which were pre-processed using CLAHE before being supplied to the VGG16 pre-trained CNN. Hayati et al. [32] showed that the average accuracy of the obtained results with pre-processed images is very competitive for pre-trained VGG16, InceptionV3, and EfficientNet CNN models versus conventional diabetic retinopathy images. The study proposed by Hussein et al. [28] used pre-processed X-ray images and pre-trained VGG 19; the architecture outperforms traditional methods by roughly 20% in terms of accuracy.

Compatibility: Through hybrid models, we found that the default SoftMax classifier did not improve performance. Replacing classifiers with MLs demonstrated an increase in model accuracy. Clear compatibility between the pre-trained CNN and the SVM algorithm was identified. Gautam et al. [33] proposed using the InceptionResNetV1, EfficientNetB7, and DenseNet121 pre-trained CNNs combined with SVM for lung carcinoma classification using histopathological images. The same combination was performed by Karuppasamy et al. [34] for classifying histopathology images of breast cancer. Therefore, this hybrid architecture proves compatible with histopathological images, and it was detected on the private dataset. Comparisons with state-of-the-art methods in Table 5 have been added; the proposed studies supplied deep learning (DL) models with pre-processed images.

Generalizability: For different DL architectures, the pre-processing step improved the models’ ability to generalize to unseen test images, as shown in our experiments. This improvement was especially noticeable when the training images were limited, helping the models adapt more effectively to variations in new images. As a result, this method not only increased accuracy but also reduced overfitting, making the models more robust in real-world applications.

Limitations: Our model tends to misclassify instances of OS vs. OS and CAF classes. The lower accuracy recorded for the binary classification of these classes indicates a need for further investigation. The misclassifications indicate intra-class similarities between the features of these classes and the CAF class, which led to confusion in the deep learning models. Deep learning models often require extensive computational time; with the L4 GPU hardware accelerator in Google Collaboratory, all proposed models exceed two hours. The limited number of samples from the private dataset is due to the number of zebrafish studied in accordance with the approved experiment. Variations in staining intensity or color balance can introduce confounding factors in image-based analysis; interpretability is considered a significant limitation, as long as the model’s predictions usually correspond to distinctive histopathological features, including, for example, the epithelial integrity, the distribution of goblet cells, etc., while the precise morphological factors that drive these classifications are still not yet defined. In the classification process, ensemble learning methods—such as bagging, boosting, and stacking—that combine predictions from multiple models to enhance performance were not utilized. Instead, the data was split into 70/30, 80/20, and 75/25 train–test ratios, and the best result was retained.

## 5. Conclusions

Histopathological images have a fundamental role in diagnostic pathology. The novelty of the proposed study is analyzing and modifying the content of intestine histopathological images with OF versus OS and TB/CAF and classifying them with deep learning models with different architectures. In addition to this, the use of the original dataset results in lower accuracy compared to studies that utilize public datasets. To determine the optimal classification between the studied classes, we enhanced all images using the CLAHE pre-processing method. This technique provides better results (accuracy of 84.6%) compared with the classification performed on raw images (accuracy of 78.4%). Out of four DL models and three ML algorithms, the hybrid models Xception and SVM provide the best results for all studied classes. Future work may include a large collection of histopathological images with different types of oxidative stress and performing predictions with the new DL models.

## Figures and Tables

**Table 1:** Summary of results obtained with pre-trained Xception CNN.

Xception	ML	Raw Images			Pre-Processed Images		
		Accuracy	F1-Score	MCC	Accuracy	F1-Score	MCC
Control vs. OS	SoftMax	0.647	0.570	0.238	0.783	0.714	0.542
	RF	0.617	0.693	0.153	0.618	0.533	0.173
	KNN	0.532	0.667	0.095	0.601	0.714	0.591
	SVM	0.705	0.744	0.277	0.784	0.775	0.574
OS vs. OS and TB	SoftMax	0.538	0.390	0.120	0.620	0.523	0.257
	RF	0.6	0.523	0.221	0.717	0.715	0.435
	KNN	0.525	0.495	0.387	0.667	0.522	0.35
	SVM	0.784	0.775	0.547	0.846	0.793	0.676
OS vs. OS and CAF	SoftMax	0.689	0.512	0.384	0.620	0.349	0.582
	RF	0.609	0.595	0.217	0.666	0.662	0.358
	KNN	0.574	0.571	0.164	0.620	0.349	0.582
	SVM	0.701	0.627	0.405	0.816	0.818	0.636

**Table 2:** Summary of results obtained with custom-built CNN.

Custom-Built	Machine Learning	Raw Images			Pre-Processed Images		
		Accuracy	F1-Score	MCC	Accuracy	F1-Score	MCC
Control vs. OS	SoftMax	0.627	0.578	0.285	0.696	0.415	0.094
	RF	0.618	0.361	0.127	0.686	0.5	0.323
	KNN	0.5392	0.5436	0.0839	0.578	0.566	0.189
	SVM	0.775	0.742	0.543	0.803	0.7142	0.591
OS vs. OS and TB	SoftMax	0.654	0.716	0.312	0.679	0.719	0.325
	RF	0.59	0.61	0.177	0.684	0.706	0.364
	KNN	0.692	0.707	0.383	0.709	0.736	0.414
	SVM	0.744	0.787	0.465	0.81	0.824	0.618
OS vs. OS and CAF	SoftMax	0.54	0.672	**0.257**	0.6322	0.6002	0.2812
	RF	0.5287	0.5272	0.0507	0.689	0.6869	0.3989
	KNN	0.5402	0.5394	0.075	0.5862	0.5339	0.2881
	SVM	0.6092	0.6	0.2489	0.6782	0.6727	0.3859

**Table 3:** Summary of results obtained with Vision Transformer.

ViT	Machine Learning	Raw Images			Pre-Processed Images		
		Accuracy	F1-Score	MCC	Accuracy	F1-Score	MCC
Control vs. OS	SoftMax	0.725	0.731	0.451	0.745	0.75	0.495
	RF	0.676	0.629	0.343	0.706	0.7	0.412
	KNN	0.598	0.4476	0	0.63	0.553	0.243
	SVM	0.745	0.729	0.491	0.761	0.725	0.514
OS vs. OS and TB	SoftMax	0.721	0.713	0.442	0.794	0.792	0.59
	RF	0.686	0.704	0.371	0.696	0.699	0.392
	KNN	0.667	0.691	0.331	0.692	0.692	0.385
	SVM	0.775	0.758	0.548	0.755	0.779	0.508
OS vs. OS and CAF	SoftMax	0.696	0.667	0.391	0.7	0.757	0.478
	RF	0.657	0.667	0.313	0.676	0.653	0.351
	KNN	0.647	0.633	0.293	0.709	0.7	0.422
	SVM	0.714	0.686	0.375	0.759	0.772	0.522

**Table 4:** Summary of results obtained with autoencoder CNN.

Autoencoder	Machine Learning	Raw Images			Pre-Processed Images		
		Accuracy	F1-Score	MCC	Accuracy	F1-Score	MCC
Control vs. OS	SoftMax	0.632	0.2	0.259	0.647	0.645	0.260
	RF	0.623	0.344	0.118	0.647	0.645	0.260
	KNN	0.525	0.362	0	0.721	0.704	0.471
	SVM	0.735	0.697	0.469	0.794	0.756	0.756
OS vs. OS and TB	SoftMax	0.644	0.726	0.305	0.813	0.732	0.611
	RF	0.609	0.695	0.216	0.784	0.766	0.567
	KNN	0.6782	0.672	0.385	0.736	0.747	0.47
	SVM	0.759	0.741	0.515	0.804	0.783	0.604
OS vs. OS and CAF	SoftMax	0.551	0.545	0.093	0.686	0.652	0.366
	RF	0.678	0.739	0.352	0.706	0.674	0.406
	KNN	0.593	0.648	0.171	0.686	0.652	0.366
	SVM	0.714	0.714	0.419	0.717	0.716	0.433

**Table 5:** Compositions with cutting-edge papers.

Reference/Years	Datatset	DL Model	Accuracy
Hussein et al./2022 [28]	Radiological Society of North America.	VGG 19	70%
Karuppasamy et al./2024 [34]	BreaKHis	VggNet-16	83%
	Sultan Qaboos University Hospital (SQUH)	ResNet-50	84%
Stefano et al./2025 [35]	CBIS-DDSM	EfficientNetB6	76.24%
Our method	Own dataset	Xception and SVM	84.6%

## Data Availability

The datasets used and analyzed during the current study are available from the corresponding author on request. The data are not publicly available due to legal or ethical reasons.
